# Signaling Role of Glutamate in Plants

**DOI:** 10.3389/fpls.2019.01743

**Published:** 2020-01-24

**Authors:** Xue-Mei Qiu, Yu-Ying Sun, Xin-Yu Ye, Zhong-Guang Li

**Affiliations:** ^1^School of Life Sciences, Yunnan Normal University, Kunming, China; ^2^Engineering Research Center of Sustainable Development and Utilization of Biomass Energy, Ministry of Education, Kunming, China; ^3^Key Laboratory of Biomass Energy and Environmental Biotechnology, Yunnan Province, Yunnan Normal University, Kunming, China

**Keywords:** environmental stress, glutamate receptor, glutamate signalling, response and adaptation, root architecture, seed germination

## Abstract

It is well known that glutamate (Glu), a neurotransmitter in human body, is a protein amino acid. It plays a very important role in plant growth and development. Nowadays, Glu has been found to emerge as signaling role. Under normal conditions, Glu takes part in seed germination, root architecture, pollen germination, and pollen tube growth. Under stress conditions, Glu participates in wound response, pathogen resistance, response and adaptation to abiotic stress (such as salt, cold, heat, and drought), and local stimulation (abiotic or biotic stress)-triggered long distance signaling transduction. In this review, in the light of the current opinion on Glu signaling in plants, the following knowledge was updated and discussed. 1) Glu metabolism; 2) signaling role of Glu in plant growth, development, and response and adaptation to environmental stress; as well as 3) the underlying research directions in the future. The purpose of this review was to look forward to inspiring the rapid development of Glu signaling research in plant biology, particularly in the field of stress biology of plants.

## Introduction

In animal system including human body, glutamate (Glu), also known as α-aminoglutaric acid, is a key excitatory neurotransmitter. Glu mediates neural signaling transduction in the synapses, participates in cognition, learning, memory, and other physiological and pathological processes (Forde, 2014; Reiner and Levitz, 2018; López-Bucio et al., 2019). Glu as a signaling molecule in animal system has been found for more than 50 years (López-Bucio et al., 2019). In presynaptic cells, Glu usually exerts its signaling role by two types of receptors, namely ionotropic glutamate receptors (iGluRs) and metabolic glutamate receptors (mGluRs). The later also known as G-protein coupled receptors (Forde, 2014; Reiner and Levitz, 2018; López-Bucio et al., 2019). The iGluRs can form ion (such as Ca^2+^, Na^+^) channels, which can be directly activated by their agonist Glu. The mGluRs can activate ion channels *via* coupling to G-protein signaling system by the following signaling pathways. 1) Ion channels can be directly activated by γ, β subunits of the G-protein; or 2) indirectly activated through triggering second messengers (such as inositol triphosphate: IP_3_; reactive oxygen species: ROS; nitric oxide: NO) (Brosnan and Brosnan, 2013; Reiner and Levitz, 2018). These indicate the signaling crosstalk between Glu and other signaling molecules in signaling transduction in plants.

It is well known that Glu is a protein amino acid, which is a precursor of the synthesis of proteins and polypeptides. Besides, Glu also is a common precursor of many organic compounds. These organic compounds include protein amino acids (glutamine: Gln; proline: Pro; arginine: Arg; and histidine: His), non-protein amino acid (γ-aminobutyric acid, GABA), antioxidant tripeptide (glutathione, GSH), heme, chlorophyll, and so forth (Brosnan and Brosnan, 2013; Reiner and Levitz, 2018). In addition, Glu has the following features: chemical stability, metabolic generation and easy removal (interconversion with α-ketoglutarate), negative charge (at physiological pH value), and acidic amino acid [due to its two carboxyl groups (α- and γ-carboxyl) and one amino group]. Therefore, Glu is a multifunctional (at least metabolite and signaling molecule) amino acid (Brosnan and Brosnan, 2013; Reiner and Levitz, 2018).

Recently, in plants, in addition to above-mentioned functions, Glu is found to emerge as a novel signaling role in many physiological processes. These processes include seed germination (Kong et al., 2015), root architecture (Forde, 2014; López-Bucio et al., 2019), pollen germination and pollen tube growth (Michard et al., 2011; Wudick et al., 2018), wound response and pathogen resistance (Manzoor et al., 2013; Mousavi et al., 2013; Nguyen et al., 2018; Toyota et al., 2018; Jin et al., 2019), and response and adaptation to abiotic stress (Cheng et al., 2018; Zheng et al., 2018; Li H. et al., 2019; Li Z. et al., 2019; Philippe et al., 2019). In addition, Glu can act as a long-distance signaling transducer among cells, tissues, organs, and even the whole plants by the crosstalk with Ca^2+^, ROS, and electrical signaling (Mousavi et al., 2013; Nguyen et al., 2018; Toyota et al., 2018). Numerous studies have showed that Glu usually exerts signaling role by its receptors, that is, glutamate receptors (GLRs), similar to iGluRs in animals (Lam et al., 1998; Wudick et al., 2018; López-Bucio et al., 2019). In plants, GLRs are at least classified into three clades: clade I (GLRs 1.1–1.4), clade II (GLRs 2.1–2.9), and clade III (GLRs 3.1–3.7) (Lam et al., 1998; Weiland et al., 2016; Wudick et al., 2018; López-Bucio et al., 2019). In this review, in view of the current opinion on Glu signaling in plants, the following knowledge was updated and discussed. 1) Glu metabolism; 2) signaling role of Glu in plant growth, development, and response and adaptation to environmental stress, as well as 3) the underlying research directions in the future was discussed. The purpose of this review was to look forward to exciting the rapid development of Glu signaling research in the plant biology, particularly in the field of stress biology of plants.

## Metabolism and Homeostasis of Glutamate in Plants

**Figure 1:**
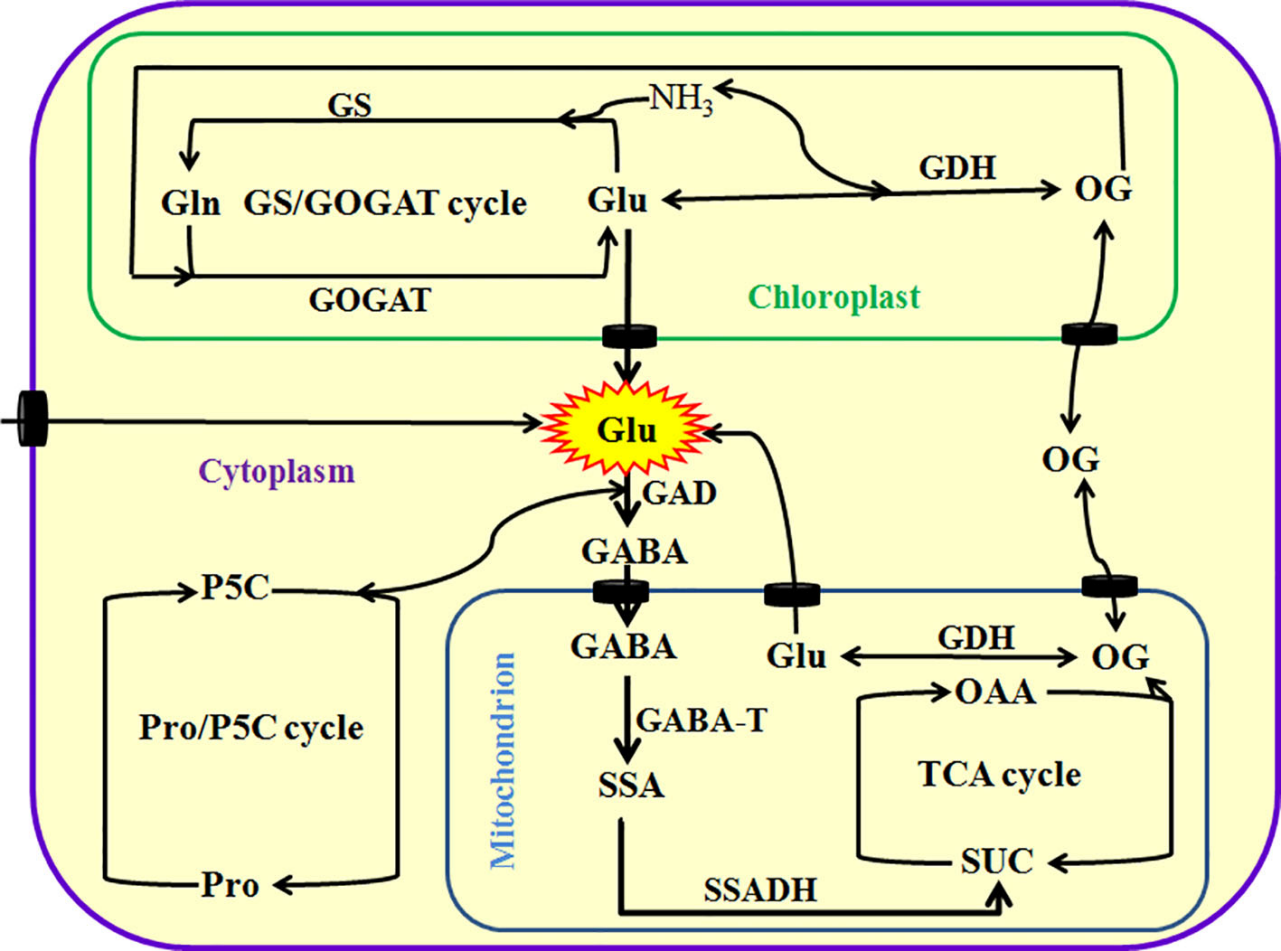
Metabolism of glutamate (Glu) in plants. Glu can be directly or indirectly synthesized by glutamine synthetase (GS)/glutamine-α-oxoglutarate transaminase (GAGOT) cycle, and glutamate dehydrogenase (GDH) in the chloroplasts, as well as proline (Pro)/pyrroline 5-carboxylate (P5C) cycle in the cytoplasm in plants. In addition, Glu can be exported into the cytoplasm by Glu transporters from chloroplasts or mitochondria, and then catabolized to γ-aminobutyric acid (GABA) by a key enzyme Glu decarboxylase (GAD). Subsequently, GABA is imported into the mitochondria by its transporters and converted into succinic semialdehyde (SSA) by GABA transaminase (GABA-T), and then produces succinate (SUC) by the catalysis of succinate semialdehyde dehydrogenase (SSADH), eventually entering into tricarboxylic acid (TCA) cycle. TCA cycle can produce 2-oxoglutarate (OG, also known as α-ketoglutarate), which in turn is converted into Glu by GDH or exported into the cytoplasm by its transporters. OAA, oxaloacetic acid.

As mentioned above, Glu plays a very important role in plant growth, development, and response and adaptation to environmental stress. In plants, Glu can be principally synthesized *via* glutamine synthetase (GS)/Glu synthase (also known as Gln-α-ketoglutarate aminotransferase, GOGAT) cycle in the chloroplasts of photosynthetic tissue or non-photosynthetic tissue plastids and Glu dehydrogenase (GDH) in the mitochondria or cytoplasm. They regulate the homeostasis of Glu, Gln, 2-oxoglutarate (GO), and ammonia (NH_3_) in plant cells. In addition, plants also can produce Glu by Pro/pyrroline 5-carboxylate (P5C) cycle and transamination, which are alternative pathways (Brosnan and Brosnan, 2013; Seifi et al., 2013; Hildebrandt et al., 2015; Majumdar et al., 2016; Liu and von Wirén, 2017; **Figure 1**). These pathways not only insure the timely supply of Glu from nitrate reduction, but also maintain ammonia homeostasis in plant cells, which prevents from the toxic action of ammonia. Excessive ammonia (NH_3_) is toxic to plant cells mainly by interfering with energy metabolism (namely eliminating proton motive force by binding H^+^ to form ammonium) and/or disrupting pH balance (Brosnan and Brosnan, 2013; Hildebrandt et al., 2015; Liu and von Wirén, 2017). Also, Arg and His can separately synthesize Glu by intermediates, N-formimino Glu (Brosnan and Brosnan, 2013; Hildebrandt et al., 2015; Liu and von Wirén, 2017).

In addition to its anabolism, Glu in the mitochondria can be exported into cytoplasm by Glu transporters, and then degraded to GABA by Glu decarboxylase (GAD). Subsequently, the GABA is imported into mitochondria by GABA permease locating on the mitochondrial endomembrane, which in turn is converted into succinic semialdehyde (SSA) by GABA transaminase (GABA-T). Afterward, SSA dehydrogenase (SSADH) catalyzes the conversion of SSA to succinate (SUC, a major source for tricarboxylic acid cycle), which in turn enters into tricarboxylic acid (TCA) cycle, further regulating redox balance and energy metabolism (Hildebrandt et al., 2015; Liu and von Wirén, 2017; **Figure 1**). This process is known as GABA shunt (Fait et al., 2008; Michaeli and Fromm, 2015; Bor and Turkan, 2019). GABA shunt not only is a bridge linking carbon and nitrogen metabolism (and even secondary metabolism), but also plays a very important role in energy, redox, carbon/nitrogen, and Glu/GABA balance (Fait et al., 2008; Michaeli and Fromm, 2015; Bor and Turkan, 2019). In addition, GABA can trigger other signaling molecules (such as Ca^2+^, reactive oxygen species, nitric oxide, and ethylene) by binding with its putative receptors, which in turn regulating polyamine metabolism and plant growth (such as pollen tube growth and development) (Fait et al., 2008; Michaeli and Fromm, 2015; Bor and Turkan, 2019).

Besides, Glu can be taken up directly by the roots, and then transported among tissues or organs through xylem and phloem. In general, the redistribution of Glu can be achieved by transporters locating in the plasma membrane. In *Arabidopsis*, the Glu transporters include cationic *amino* acid transporters (CAT) family (amino acid-polyamine-choline facilitator, APC superfamily), γ-aminobutyric acid (GABA) permease-related (APC superfamily), and amino acid transporter family (ATF superfamily) (Fischer et al., 1998; Yang et al., 2014; Errasti-Murugarren et al., 2019). Glu redistribution can alter plant growth and development, which in turn improve the resistance of plants to adverse environments (Yang et al., 2014). Therefore, these transporters play a very important role in Glu uptake, homeostasis, and signaling in plants.

Generally, Glu is maintains homeostasis in plant cells by above biosynthesis and catabolism pathways. In different tissues, organs, and subcellular organelles, Glu has been found to have different concentrations. In *Arabidopsis thaliana* leaves, the amount of Glu remains about 1 μmol g^−1^ fresh weight (FW), but in phloem cells, Glu content reaches 50 mM (Geiger et al., 1998; Forde and Lea, 2007; Hunt et al., 2010). When the phloem cells were wounded by herbivores or external forces, Glu will pour into apoplastic spaces (cell wall), initiating Glu signaling-centered transduction pathways (Toyota et al., 2018). In addition, the different concentrations of Glu were observed in various plant species, such as tobacco (3~4 μmol g^−1^ FW, Geiger et al., 1998), potato (1~1.3 μmol g^−1^ FW, Urbanczyk-Wochniak et al., 2005), tomato (2.32 μmol g^−1^ FW, Roessner-Tunali et al., 2003), and spinach (2 μmol mg^−1^ chlorophyll, Riens et al., 1991) leaves. Similarly, the concentrations of Glu were monitored in phloem cells of broad bean (7.4 mM), alfalfa (12.7 mM), red clover (13.8 mM) (Sandstrom and Pettersson, 1994), while 1 mM in leaf apoplasts of spinach (Lohaus and Heldt, 1997) and *Arabidopsis* (Toyota et al., 2018). In short, the concentrations of Glu vary in plant species, but that of Glu in phloem cells are higher than that of extracellular spaces, which might be the basis for Glu as signaling molecule in plants.

## Glutamate Signaling in Plant Growth and Development

### Seed Germination

For seed plants, seed germination is the first and a key step in plant life cycle, which is an important basis of successful plant cloning and propagation. In general, seed germination is tightly regulated by the synergistic action of endogenous (mainly phytohormones such as abscisic acid, ABA; gibberellin, GA; ethylene, ETH) and exogenous signals (such as light, temperature, water, and oxygen) (Née et al., 2017; Penfield, 2017; Duermeyer et al., 2018; Prakash et al., 2019). Because seed germination is sensitive and fragile to environmental stress, which has become an important research mode of plant signaling transduction. This signaling mode is involved in numerous signaling molecules (Ca^2+^; ROS; nitric oxide: NO; hydrogen sulfide: H_2_S; and plant hormones) and their interactions (Née et al., 2017; Penfield, 2017; Duermeyer et al., 2018; Prakash et al., 2019).

Numerous studies have illustrated that exogenous (cell wall) Ca^2+^ takes part in seed germination *via* across membrane into the cytosol, which is mediated by Ca^2+^ channels (Gong et al., 1997; Verma et al., 2019). In *A. thaliana*, the gene expression of GLR homolog3.5 (AtGLR3.5, constituting Ca^2+^ channels) was significantly up-regulated in germinating seeds, which in turn increased cytosolic Ca^2+^ concentration, eventually stimulating seed germination *via* counteracting the inhibiting effect of ABA (Kong et al., 2015). Adversely, inhibiting expression of AtGLR3.5 blocked Ca^2+^ increase in cytosol, followed by delaying germination and increasing ABA sensitivity. In addition, the overexpression of AtGLR3.5 lowered the sensitivity of seeds to ABA and reduced the time of germination (early germinating). Also, Ca^2+^ could inhibit the gene expression of ABSCISIC ACID INSENSITIVE4 (ABI4, a transcription factor), which is involved in ABA response in seeds (Kong et al., 2015). These results provide a molecular evidence that the Ca^2+^ influx mediated by AtGLR3.5 promotes seed germination *via* relieving the inhibitory effects of ABA by inhibiting ABI4 action.

### Root Architecture

The plant root system accounts for 80% of the whole plant, which not only anchors the plant into the soil, but also finds, uptakes, and transports nutrient. In general, the root architecture is regulated by a various of signaling molecules, such as Ca^2+^, ROS, NO, H_2_S, Glu, and plant hormones (ABA, auxin, and cytokinin), as well as their crosstalk (Chen and Kao, 2012; Mei et al., 2017; López-Bucio et al., 2019). Recently, Singh and Chang (2018) reported that, in model plant *Arabidopsis*, iGluR competitive antagonists [6-cyano-7-nitroquinoxaline-2,3-dione (CNQX) and 6,7-dinitroquinoxali-ne-2,3-dione acted (DNQX)] dramatically inhibited the growth of primary root and the density of lateral root by impairing root meristem size. On the contrary, the treatment with iGluR agonist Glu recovered the growth of *Arabidopsis* roots. In addition, a low amount of Ca^2+^ chelator [ethylene glycol-bis(b-aminoethylether)-N,N,N΄,N΄-tetraacetic acid, EGTA] drastically inhibited the root elongation of *Arabidopsis*, while this inhibition was partially recovered by Glu (Singh and Chang, 2018). The further experiments showed that 1-N-naphthylphthalamic acid (NPA, a polar auxin transport inhibitor) inhibited the growth of primary root and reduced the density of lateral roots, whereas this negative effect of NPA was rescued by exogenous Ca^2+^ and Glu, respectively (Singh and Chang, 2018), indicating that synergistic action of Ca^2+^, auxin, and Glu signaling in the structure of root architecture in *Arabidopsis*. Similarly, Glu inhibited the growth of primary root and stimulated the outgrowth of lateral roots, forming a shorter and more branched root system in *Arabidopsis* (Walch-Liu et al., 2006). These findings show that GLRs, iGluR-like channels, take part in the development of root architecture by regulating meristem maintenance in *Arabidopsis*.

In addition, the further pharmacological, electrophysiological, and molecular genetic approaches also confirm that GLRs participate in the establishment of root architecture in *Arabidopsis* by interaction with Ca^2+^, mitogen activated protein kinase (MAPK), ROS, and auxin (Forde, 2014; López-Bucio et al., 2019). In *Arabidopsis*, the knockout mutant plants of *AtGLR3.2* or *AtGLR3.4* genes lead to the strong increase in the number of lateral root primordia, but no significant effect on the number of visible lateral roots, suggesting the *AtGLR3.2* and *AtGLR3.4* regulate the initiation of lateral roots (Vincill et al., 2013). In addition, the AtGLR3.2/AtGLR3.4 receptors may locate on the surface of the phloem cells to regulate the root architecture by mediating Ca^2+^ signaling in response to extracellular amino acids including Glu (Vincill et al., 2013). Similarly, in rice (*Oryza sativa*), the *OsGLR3.1* knockout mutant plants also show a short root, and the further experiments indicate that *OsGLR3.1* positively regulates cell proliferation and survival in the root apical meristem (Li et al., 2006).

Also, MAPKs and MAPK phosphatases (MKPs) play a very important role in the plant signaling transduction, which regulate most biological processes including root formation. In *Arabidopsis*, Glu treatment results in the inhibition of root growth and the curvature of the root tip in wild-type (Col-0) plants, whereas the Glu-induced growth inhibition and curvature were restored in mutant MPK6 kinase (*mpk6*) seedlings, but aggravated in mutant *mkp1* seedlings (Forde, 2014; López-Bucio et al., 2019). These observations imply that the both enzymes (mpk6 and mkp1) participate in the construct of root architecture in *Arabidopsis* by Glu signaling pathway.

### Pollen Germination and Pollen Tube Growth

In higher plants, pollen germination and pollen tube growth are basis for achieving double fertilization, which is a key stage in the whole life cycle of plants. The process needs to perceive and integrate all kinds of signaling molecules, such as Ca^2+^, ROS, NO, H_2_S, and plant hormones, eventually forming a sophisticated signaling network controlling pollen germination and pollen tube growth (Dresselhaus and Franklin-Tong, 2013; Dresselhaus et al., 2016; Zhou and Dresselhaus, 2019).

In general, Ca^2+^ plays a central role in pollen germination and pollen tube growth, and an increase in cytosolic free calcium concentration ([Ca^2+^]cyt) is a fundamental signaling transduction event (Steinhorst and Kudla, 2013; Zheng et al., 2019). However, the formation mechanism of Ca^2+^ gradient (signaling) in pollen germination and pollen tube growth is not completely clear. Michard et al. (2011) have found that, in both tobacco and *Arabidopsis*, GLRs can form Ca^2+^ channels and mediate Ca^2+^ influx across the plasma membrane, which in turn modulates apical [Ca^2+^]cyt gradient and consequently regulates pollen tube growth and morphogenesis. Adversely, iGluR antagonists CNQX, DNQX, and DL-2-amino-5-phosphonopentanoic acid (AP-5) show an inhibitory effect on Ca^2+^ signaling and pollen tube growth (Michard et al., 2011). In addition, the *AtGLR1.2* and *AtGLR3.7* mutants can weaken Ca^2+^ signaling in pollen tube, the *AtGLR1.2* mutants show abnormally deformed tips and tubes, while the *AtGLR3.7* mutant tubes grow slower than wild-type (Michard et al., 2011). These results show a novel signaling mechanism in pollen germination and tube growth between male gametophyte and pistil tissue, which is similar to animal nervous systems based on amino acid–mediated communication.

In addition, GLRs not only are activated by amino acids (Glu; D-serine: D-Ser; Arg; glycine: Gly; isoleucine: Ile; Pro; serine: Ser; threonine: Thr; and tryptophan: Try) in *Arabidopsis* (Qi et al., 2006; Michard et al., 2011), but also CORNICHON HOMOLOG (CNIH) proteins (*At*CNIHs) done (Wudick et al., 2018). The specific *At*CNIHs can enhance the activity of GLRs (mainly AtGLR3.3) by regulating their sorting, trafficking, location, and quantity without any ligands, which in turn contribute to cytosolic Ca^2+^ homeostasis, thus regulating pollen germination and pollen tube growth (Wudick et al., 2018).

## Glutamate Signaling in Plants Response and Adaptation to Environmental Stress

In addition to seed germination, root architecture, and pollen germination and tube growth mentioned above, Glu as a signaling molecule is also involved in the response and adaptation to salt, cold, heat, drought, pathogen, and wound stress (**Table 1**), which are discussed as follows in detail.

**Table 1:** Glutamate and its receptors response to environmental stress in plants.

Plant species	Stress	GLRs	Mediators	References
*Arabidopsis*	Salt	AtGLR3.4, AtGLR3.7	Ca^2+^ signaling, SOS3, SOS2, and SOS1	Cheng et al., 2018
	Salt	AtGLR3.7	Ca^2+^ signaling, 14-3-3 proteins	Wang et al., 2019
*Arabidopsis*	Cold	AtGLR1.2, AtGLR1.3, and AtGLR3.4	JA, CBF/DREB1	Zheng et al., 2018
*Solanum lycopersicum*	Cold	GLR3.3 and GLR3.5	H_2_O_2_, redox homeostasis	Li H. et al., 2019
*Zea mays*	Heat	GLRs	Ca^2+^ signaling	Li Z. et al., 2019
*Vicia faba*	Drought	GLR3.5	Ca^2+^ signaling, ABA, and CDPK6	Yoshida et al., 2016
*Medicago truncatula*	Drought	GLR2.5, GLR2.7, GLR2.8, GLR2.9, GLR3.1, GLR3.2, GLR3.3, GLR3.4, GLR3.6, GLR3.7	NO, ABA	Philippe et al., 2019
*Brassica napus*	Drought	ND	Ca^2+^ signaling, SA, proline	La et al., 2019
*Pyrus bretschneideri*	Pathogen	GLRs	PR1, CHI4, PAL, GABA, and arginine	Jin et al., 2019
*Solanum lycopersicum*	Pathogen	GLR1.1, GLR1.2, GLR2.1, GLR2.4, GLR2.5, GLR2.6, GLR3.1, GLR3.2, GLR3.3	GABA, PR1, PR2, PR3, PR4, methionine, asparagine, phenylalanine, histidine, lysine, and arginine	Sun et al., 2019
*Arabidopsis*	Pathogen	GLR3.3	NO, ROS, OGs	Li et al., 2013; Manzoor et al., 2013
*Arabidopsis*	Wound	GLRs	Ca^2+^ signaling	Toyota et al., 2018
*Arabidopsis*	Wound	GLR3.1, GLR3.2, GLR3.3	Ca^2+^ signaling	Nguyen et al., 2018
*Arabidopsis*	Wound	GLRs	Ca^2+^ signaling, JA	Mousavi et al., 2013; Wasternack, 2019

ABA, abscisic acid; CBF/DREB1, C-repeat binding factor/dehydration responsive element binding1; CDPK, calcium dependent protein kinase; CHI, chitinase; GABA, γ-aminobutyric acid; GLR, glutamate receptors; JA, jasmonate; NO, nitric oxide; OGs, oligogalacturonides; PA, phenylalanine ammonialyase; PR, pathogenesis-related proteins; ROS, reactive oxygen species; SOS, salt overly sensitive proteins.

ND, no detection.

### Salt Stress

Salt stress commonly triggers early (osmotic phase) and late (ionic phase) stress phase. The former refers to the rapid production of osmotic stress within several minutes due to the quick decline in extracellular water potential, while the latter is ion and oxidative stress as well as nutrient imbalance as a result of accumulation of Na^+^ and Cl^−^ with time passing by. The symptoms of late stress phase can be observed within several days (Deinlein et al., 2014; Muchate et al., 2016; Saddhe et al., 2019). Generally, plant response and adaptation to salt stress is implicated in a sophisticated signaling interaction network based on Ca^2+^ signaling. Therefore, Ca^2+^/calmodulin-centered Ca^2+^ messenger system plays a crucial role in the response and adaptation to salt stress in plants (Deinlein et al., 2014; Muchate et al., 2016; Saddhe et al., 2019).

As discussed above, seed germination is sensitive and fragile to environmental stress including NaCl stress, and Ca^2+^ and ABA regulate seed germination under NaCl stress. In *A. thaliana*, the results of Cheng et al. (2018) showed that, compared with the wild-type plants, the *AtGLR3.4-1* and *AtGLR3.4-2* mutant plants were more sensitive to NaCl in both seed germination and post-germination seedling growth. In addition, in wild-type seedlings, NaCl triggered a striking increase in [Ca^2+^]_cyt_, but this increase was blocked by DNQX (GLR antagonist); whereas in both *AtGLR3.4-1* and *AtGLR3.4-2* mutants NaCl-triggered increase in [Ca^2+^]_cyt_ was impaired (Cheng et al., 2018). Also, under NaCl stress, both mutants showed a lower expression of salt overly sensitive (*SOS3), SOS2*, and *SOS1*, as well as more accumulation of Na^+^ than wild-type seeds (Cheng et al., 2018). In addition to these, compared with the wild-type plants, the both mutants are more sensitive to ABA, while overexpression of *AtGLR3.4* was more tolerant to ABA. Also, the content of ABA in both mutants and wild-type plants was no significant difference, accompanied by lower expression of *ABI3* and *ABI4* in *AtGLR3.4* mutants under NaCl stress (Cheng et al., 2018). Similarly, in *Arabidopsis*, besides AtGLR3.4, AtGLR3.7 also is involved in the regulation of seed germination under NaCl stress (Cheng et al., 2016). Recently, Wang et al. (2019) also found that AtGLR3.7 interaction with 14-3-3 proteins participated in salt stress response in *Arabidopsis* by affecting calcium signaling pathways. The data imply that Ca^2+^ influx mediated by AtGLRs (mainly AtGLR3.4 and AtGLR3.7) regulates seed germination under salt stress.

### Cold Stress

In plants, numerous studies have demonstrated that plasma membrane is the primary site perceiving cold stress. The perception of cold stress by plasma membrane can activate Ca^2+^ channels and triggers Ca^2+^-centered signaling transduction pathways, eventually responding and adapting to cold stress (Miura and Furumoto, 2013; Zhu, 2016; Friedrich et al., 2018; Raju et al., 2018; Ding et al., 2019). In *Arabidopsis* seedlings, *AtGLR1.2 and AtGLR1.3* mutants were sensitive to cold stress, while treatment with jasmonate (JA, a plant hormone) alleviated the sensitivity of *AtGLR1.2 and AtGLR1.3* mutants to cold stress. In turn, the overexpression of AtGLR1.2 or AtGLR1.3 could improve the tolerance of mutants to cold stress by synthesizing endogenous JA (Zheng et al., 2018). In addition, under cold stress, both mutants showed a lower expression of C-repeat binding factor/dehydration responsive element binding1 (CBF/DREB1) compared with the wild-type plants, while the expression of *AtGLR1.2-OE* [*AtGLR1.2-GFP* (green fluorescence protein)] and *AtGLR1.3-OE* [*AtGLR1.2-GFP*] was upregulated in transgenic plants (Zheng et al., 2018). The further experiments confirmed that AtGLR1.2 and AtGLR1.3 independently exerted similar role in JA-induced cold tolerance (Zheng et al., 2018). Recently, in tomato (*Solanum lycopersicum*), Li H. et al. (2019) reported that cold acclimation at 12°C upregulated the expression of *GLR3.3* and *GLR3.5*, which in turn increased the resistance of tomato plants to a subsequent chilling stress at 4°C. On the contrary, the plants silenced *GLR3.3* or/and *GLR3.5* or supplemented DNQX all reversed the chilling tolerance induced by cold acclimation compared with the wild types. In wild types, Glu (as a GLR agonist) pretreatment increased the chilling tolerance, but *GLR3.3-* or *GLR3.5*-silenced or cosilenced plants failed (Li H. et al., 2019). In addition, the gene silence or DNQX addition inhibited the gene expression of *respiratory burst oxidase homolog1* (*RBOH1*) and its activity, further reduced the level of apoplastic H_2_O_2_ and the GSH/oxidized glutathione (GSSG) ratio, finally led to the decline in chilling tolerance (Li H. et al., 2019). However, the declined chilling tolerance induced by gene silence or DNQX was rescued by H_2_O_2_ or GSH (Li H. et al., 2019). Furthermore, the tomato plants cosilenced both *RBOH1*-silenced and GSH biosynthesis genes [*γ-glutamylcysteine synthetase: GSH1* and *GSH synthetase* (*GSH2*)] also reduced the chilling tolerance, accompanying with reduced GSH/GSSG ratio. Pretreatment with DNQX had no significant effect on the GSH/GSSG ratio and the chilling tolerance in *RBOH1*-silenced plants and *GSH1*- and *GSH2*-cosilenced plants (Li H. et al., 2019). These findings indicate that plant GLRs (mainly GLR1.2, GLR1.3, GLR3.3, and GLR3.5) positively regulate the cold tolerance of plants by accumulating endogenous JA and subsequently activating the downstream CBF/DREB1 signaling pathway, or activating GLR-H_2_O_2_-GSH cascade.

Interestingly, in *Arabidopsis*, Meyerhoff et al. (2005) reported that Glu, cold, touch, and osmotic stress stimulated the expression of *AtGLR3.4 in an ABA-independent manner, but Ca*^*2+*^*-dependent fashion. Also, in transgenic plants* expressing the Ca^2+^-reporter aequorin, Glu, and cold could trigger an increase in [Ca^2+^]_cyt_, and this increase was blocked by GLRs antagonists DNQX and CNQX (Meyerhoff et al., 2005). These results show that AtGLR3.4 plays a very important role in the Ca^2+^-based, fast signaling transmission of environmental stress, further supporting the fact that GLRs regulate the cold tolerance of plants.

### Heat Stress

High temperature, above the optimal growth temperature, is a major stress factor that impacts on the whole life cycle of plants; Ca^2+^, crossing membrane into the cells *via* Ca^2+^ channels, is implicated in the development of heat tolerance in plants (Gong et al., 1997; Wahid et al., 2007; Li et al., 2012; Asthir, 2015; Li H. et al., 2019; Li Z. et al., 2019; Serrano et al., 2019). Recently, the positive effect of Glu in heat tolerance had been found by Li Z. et al. (2019), who first reported that in maize (*Zea mays* L.) seedlings treatment with Glu elevated the survival percentage of seedlings under heat stress. Also, heat tolerance induced by Glu was impaired by exogenous CaCl_2_ (may produce Ca^2+^ overloading), LaCl_3_ (plasma membrane Ca^2+^ channel blocker), EGTA (Ca^2+^ chelator), as well as trifluoperazine and chlorpromazine (calmodulin antagonists) (Li Z. et al., 2019). Similarly, the Glu-induced heat tolerance was weakened by GLR antagonist (DNQX) and its blocker (MgCl_2_) (Li Z. et al., 2019). This work has for the first time reported that Glu is able to enhance the heat tolerance of maize seedlings by activating GLRs-mediated calcium signaling, but the detailed physiological and molecular mechanisms need to be further explored in the future.

### Drought Stress

Drought stress caused by the decline in soil water potential interferes with many physiological processes at different levels, such as water uptake, transpiration, photosynthesis, seed germination, plant growth, and development (Deinlein et al., 2014; Tardieu et al., 2018; Hasanuzzaman et al., 2019; Laxa et al., 2019). Under severe conditions, drought stress lead simultaneously or successively to osmotic and oxidative stress. Therefore, the regulation of water balance by controlling stomata movement is one of the major strategies that resist and adapt to drought stress in plants (Deinlein et al., 2014; Tardieu et al., 2018; Hasanuzzaman et al., 2019; Laxa et al., 2019).

For land plants, stomata is indispensable structure to control water balance and gas exchange, its movement is regulated by a coordinated action of a series of signaling molecules, such as Ca^2+^, ROS, NO, H_2_S, and ABA (Tardieu et al., 2018; Hasanuzzaman et al., 2019; Laxa et al., 2019). In *Arabidopsis* and fava bean (*Vicia faba* L.), Glu treatment increased [Ca^2+^]_cyt_ in guard cells, which in turn led to stomata closure. Glu-induced stomata closure was abolished by EGTA (extracellular Ca^2+^ chelator), BAPTA-AM (intracellular Ca^2+^ chelator), La^3+^ (plasma membrane Ca^2+^ channel blocker), and AP-5 (a specific antagonist of GLRs) (Yoshida et al., 2016). In addition, in *Arabidopsis* ABA-deficient (*aba2-1*) and ABA-insensitive (*abi1-1* and *abi2-1*) mutants, Glu treatment also induced stomata closure, further indicating this closure is ABA-independent manner (Yoshida et al., 2016). Similarly, in slow anion channel-associated 1 (*slac1*) mutants, Glu treatment had no significant on stomata closure. Similar results could be observed in calcium-dependent protein kinase 6 (*cdpk6*) mutants, suggesting Glu induces stomata closure in a SLAC- and CDPK-dependent manner (Yoshida et al., 2016). For *GLR3.5* mutants, Glu treatment had no effect on stomata closure, further supporting the signaling function of Glu by activating GLRs-mediated Ca^2+^ signaling pathway (Yoshida et al., 2016). In addition, in *Brassica napus*, Glu treatment triggered calcium signaling (mainly calcium-dependent protein kinase), which in turn increased the synthesis of salicylic acid. Then, which enhanced drought-induced proline accumulation, thus improving drought tolerance by regulating cellular redox potential (La et al., 2019).

In a similar vein, in *Medicago truncatula* seedlings, Philippe et al. (2019) identified 29 independent *MtGLR* genes (such as MtGLR2.5, 2.7, 2.8, 2.9, 3.1, 3.2, 3.3, 3.4, 3.6, and 3.7), which were classified into four clades based on a phylogenetic analysis, and 17 of which showed specific domains consistent with iGluRs. Under drought stress, treatment with GLR competitive antagonists (AP-5 and DNQX) reduced the accumulation of NO induced by ABA, which was no significant effect on stomata closure (Philippe et al., 2019). In addition, drought stress inhibited the elongation of embryo axis, and this inhibition was deteriorated by GLR antagonists AP-5 and DNQX (Philippe et al., 2019). These results demonstrate that Glu can promote stomata closure by activating GLR-mediated Ca^2+^ signaling, which in turn generates NO in *Medicago* seedling.

### Pathogen Stress

Similar abiotic stress, biotic stress response and resistance also is involved in various signaling molecules (Ca^2+^, ROS, NO, H_2_S, and Glu) and their cross talks (Seifi et al., 2013). Glu pretreatment increased the resistant to *Penicillium expansum* in pear (*Pyrus bretschneideri*) fruit under normal or cold conditions (Jin et al., 2019). In addition, Glu also reduced the spore germination of *P. expansum both in fruit wounds and in vitro culture* (Jin et al., 2019)*. Also, Glu enhanced the activities of* β-1,3-glucanase (GLU), chitinase (CHI), phenylalanine ammonialyase (PAL), peroxidase, and polyphenol oxidase, as well as the gene expression of *pathogenesis-related proteins1 (PR1)*, *GLU*, *CHI3, CHI4, and PAL* (Jin et al., 2019). In addition to these, Glu pretreatment promote the metabolism of amino acids, especially the accumulation of Glu, GABA, and arginine (Jin et al., 2019). Similarly, in tomato fruits, Glu pretreatment could upregulate the gene expression of nine GLRs (GLR1.1, GLR1.2, GLR2.1, GLR2.4, GLR2.5, GLR2.6, GLR3.1, GLR3.2, and GLR3.3) and four PRs (PR1a, PR2a, PR3a, and PR3b), as well as the accumulation of eight amino acids (Glu; GABA; methionine: Met; asparagine: Asn; phenylalanine: Phe; His; Lys; and Arg). In turn, which induced the resistance of tomato fruits to *Botrytis cinerea* (Sun et al., 2019). In *Arabidopsis*, Manzoor et al. (2013) reported that GLRs partially mediated the increase in [Ca^2+^]_cyt_, which in turn induced the accumulation of NO and ROS, as well as the gene expression of oligogalacturonides (OGs, defense-related proteins). In addition, in wild-type (Col-0) plants, pretreatment with DNQX was sensitive to *B. cinerea* and *Hyaloperonospora arabidopsidis* (Manzoor et al., 2013). Further experiments showed that AtGLR3.3 required for the resistance against *H. arabidopsidis*, the defense gene expression and the generation of NO and ROS triggered by OGs are AtGLR3.3-dependent (Manzoor et al., 2013). The data suggest that AtGLRs (mainly AtGLR3.3) are required in elicitor/pathogen-mediated defense signaling pathways in *Arabidopsis*.

In addition, the AtGLR3.3-mediated Ca^2+^ signaling could be triggered by endogenous GSH in *Arabidopsis*, while blocked by GLR antagonists AP-5 and DNQX, but abolished in *AtGLR3.3* mutants. Meanwhile, *AtGLR3.3* mutants reduced the expression of defense genes and increased the sensitivity to the bacterial pathogen *Pseudomonas syringae* pv. *tomato* DC3000 (Li et al, 2013). Also, Glu also could induce AtGLR3.3-dependent transcription response of defense genes, but lower than that of GSH pretreatment (Li et al., 2013). These findings further support the fact that GLRs (especially AtGLR3.3) are involved in the response and adaptation to pathogen stress in plants.

### Wound Stress

Though plants have no nervous system, they can rapidly respond to environmental stimulation including wound stress by a sophisticated signaling transduction pathway. In other words, plants can sense local stimulation/signaling (such as herbivore attack, wound, drought, and high light) and transmit this information to the whole plant body, thus triggering the defense responses, systemic acquired resistance (SAR), or systemic acquired acclimation (SAA) (Choi et al., 2017; Sukhov et al., 2019; Takahashi and Shinozaki, 2019; Wasternack, 2019). Toyota et al. (2018) reported that Glu, as a wound signaling, could be rapidly released from phloem cells when *Arabidopsis* was wound by herbivores or external force. The released Glu bound to GLRs locating on the surface of the phloem cells, and then triggered an increase in [Ca^2+^]_cyt_, thus propagating long-distance, Ca^2+^-based defense response, and even spreading over the whole plant body.

Similarly, in *Arabidopsis*, wounding triggered and propagated the membrane depolarizations in a GLR-dependent manner, which resulted in the activation of defense genes. The *GLR* mutants attenuated leaf-to-leaf electrical signaling, but *GLR3.3* mutants lost the defense against herbivores (Nguyen et al., 2018). In addition, in wild-type *Arabidopsis* plants, wounding induced the membrane depolarizations, which in turn triggered [Ca^2+^]_cyt_ peak. In *GLR3.1, 3.2, 3.3*, and *3.6* mutants, Ca^2+^ propagations were weakened differently in the axial and radial distributions (Nguyen et al., 2018). The data expound the key role of GLR3.3 in the defense-related signaling (Ca^2+^ signaling and electrical signaling) propagation triggered by herbivores.

In addition, further experiments found that in *Arabidopsis* wounding-triggered Ca^2+^ signaling promoted the biosynthesis of JA (a potential regulator of defense response) by stimulating lipoxygenase and/or inhibiting phosphorylation of repressors JAV1 within the JAV1-JAZ-8-WRKY51 complex, finally activating plant defense response (Mousavi et al., 2013; Wasternack, 2019).

## Conclusion and Perspectives

**Figure 2:**
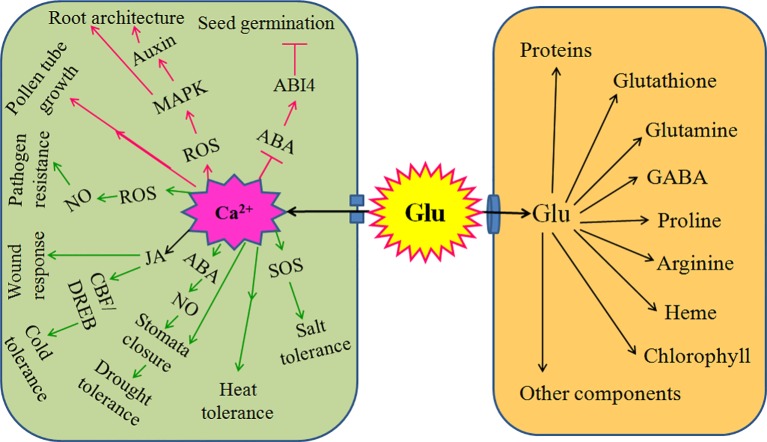
Signaling role of glutamate (Glu) in plant growth, development, and response to environmental stress. Extracellular Glu can exert its signaling role by glutamate receptor-like proteins (GLRs)-mediated calcium (Ca^2+^) signaling in plants. For plant growth and development, GLRs-mediated Ca^2+^ signaling triggered by Glu promotes seed germination by counteracting the effect of abscisic acid (ABA) and/or its receptor ABA-insensitive 4 (ABI4); constructs root architecture by crosstalk among reactive oxygen species (ROS), mitogen activated protein kinase (MAPKs), and auxin; and regulates pollen germination and pollen tube growth. In addition, for response to environmental stress, GLRs-mediated Ca^2+^ signaling triggers salt tolerance by activating salt overly sensitive (SOS) signaling pathway; induces heat tolerance; governs drought tolerance *via* interaction of ABA and nitric oxide (NO) to regulate stomata movement; improves cold tolerance by ROS-glutathione (GSH) cascade or interaction of jasmonate (JA) and C-repeat binding factor/dehydration responsive element binding (CBF/DREB) regulator pathway; responds to wound stress by JA; and initiates pathogen resistance by ROS-NO interaction. Also, extracellular Glu can import into cells and act as mediator (storage) to synthesize proteins, glutathione, glutamine, proline, gamma-aminobutyric acid (GABA), arginine, heme, chlorophyll, and other components, which in turn regulates plant growth, development, and response to environmental stress. The arrows (→) indicate facilitating effect, while the blunt lines (┬) represent inhibiting effect.

Mounting evidences have found that Glu is a novel signaling molecule, which is involved in many physiological processes. These processes include seed germination, seedling establishment, plant growth, development, senescence, and even response and adaptation to environmental stress, such as salt, cold, heat, drought, wound, and pathogen stress. In soybean crop, foliar or seed application of Glu in both greenhouse and field could enhance the antioxidant capacity by activating antioxidant enzymes catalase, peroxidase, superoxide dismutase, polyphenol oxidase, and phenylalanine ammonia lyase (Teixeira et al., 2017) and improved nitrogen metabolism and productivity (Teixeira et al., 2018). Under drought stress conditions, foliar or seed application of Glu, as stress reducer, increased Pro and relative water content, leaf and root dry weight, and plant productivity (Teixeira et al., 2020). These might be the basis of Glu-induced stress tolerance in plants. Glu not only a metabolic mediator (storage), which takes part in the biosynthesis of many organic compounds such as amino acids, GABA, proteins, chlorophyll, and so on; but also a novel signaling molecule implicating in all aspects of the life cycle of plants (Brosnan and Brosnan, 2013; Reiner and Levitz, 2018). Glu as a signaling molecule, whose functions are usually achieved by plant GLRs, which is similar to iGluRs in animals. During the process, Glu signaling usually interacts with other signaling molecules such as chemical signaling (Ca^2+^, ROS, NO) and electrical signaling, which in turn takes shape a sophisticated signaling network, eventually regulating accurately plant growth, development, and defense response (**Figure 2**).

Recently, in rice, Kan et al. (2017), using transcriptome analysis, revealed that Glu irrigation within 30 min could upregulate the expression of at least 122 genes. These genes are involved in the metabolism, transport, signal transduction, and stress responses. In addition, some transcription factor, kinase/phosphatase, and elicitor-responsive genes were induced by Glu. These results further support the signaling role of Glu in the cellular metabolism, plant growth, and defense responses.

Glu, however, as a novel signaling molecule in plants, the following open questions look forward to being uncovered in the future: 1) the detailed mechanisms of Glu-triggered abiotic and biotic tolerance, especially heat tolerance, will be illuminated at physiological, biochemical, molecular, and even omics levels. 2) Though the members and functions of GLRs have been expounded in many plant species, such as *Arabidopsis* (Lam et al., 1998), rice (Li et al., 2006), *M. truncatula* (Philippe et al., 2019), and pear (Fabrice et al., 2018), but the constitutes and functions of GLRs need to be explored and found in various plant species. 3) Glu can induce action potentials (long-distance electrical signals), which can be modulated by osmotic and salt stresses, followed by regulating growth and circumnutation in *Helianthus annuus* seedlings (Stolarz and Dziubinska, 2017). Thus imply the crosstalk between Glu and electrical signaling in plant excitability and growth movement. However, the interaction of Glu signaling with other signaling (Ca^2+^, ROS, and NO), especially H_2_S and methylglyoxal signaling, also needs to be further solved.

## Author Contributions

Z-GL conceived, designed, and wrote the manuscript. X-MQ, Y-YS, and X-YY wrote the manuscript. All authors have approved the final version of the manuscript.

## Funding

This research is supported by National Natural Science Foundation of China (31760069, 31360057).

## Conflict of Interest

The authors declare that the research was conducted in the absence of any commercial or financial relationships that could be construed as a potential conflict of interest.
